# A genetic polymorphism of the osteoprotegerin gene is associated with an increased risk of advanced prostate cancer

**DOI:** 10.1186/1471-2407-8-224

**Published:** 2008-08-06

**Authors:** Naofumi Narita, Takeshi Yuasa, Norihiko Tsuchiya, Teruaki Kumazawa, Shintaro Narita, Takamitsu Inoue, Zhiyong Ma, Mitsuru Saito, Yohei Horikawa, Shigeru Satoh, Osamu Ogawa, Tomonori Habuchi

**Affiliations:** 1 Department of Urology, Akita University School of Medicine, 1-1-1 Hondo Akita, 010-8543, Japan; 2 Department of Urology, Kyoto University Graduate School of Medicine, 54 Shogoin Kawahara, Sakyo-ku, Kyoto, 606-8507, Japan

## Abstract

**Background:**

The purpose of this study was to evaluate the role of osteoprotegerin gene *(OPG) *polymorphisms as genetic modifiers in the etiology of prostate cancer (PCa) and disease progression.

**Methods:**

Three hundred and sixty one patients with PCa and 195 normal controls were enrolled in the study, and two genetic polymorphisms, *149 T/C *and *950 T/C *in the putative promoter region of *OPG*, were genotyped.

**Results:**

There was no significant difference in the genotype frequencies between PCa patients and controls (*P *= 0.939 and 0.294 for *149 T/C *and *950 T/C *polymorphisms, respectively). However, those patients with *TC *and *TT *genotypes in the *950 T/C *polymorphism had a significantly increased risk of extraprostatic (age-adjusted odds ratio; aOR = 1.74 and 2.03 for *TC *and *TT *genotypes compared with the *CC *genotype, *P *= 0.028) and metastatic disease (aOR = 1.72 and 2.76 for *TC *and *TT *genotypes compared with the *CC *genotype, *P *= 0.009) compared with those with the *CC *genotype. In addition, analysis of the metastatic PCa patients (Stage D) showed that the presence of the *T *allele of the *OPG 950 T/C *polymorphism was an independent risk factor predicting survival by Cox proportional hazard regression analyses (*P *= 0.031).

**Conclusion:**

Progression of PCa may be influenced by an intrinsic genetic factor of the host's bone metabolism. The variant *C *allele of *950 T/C *in the *OPG *promoter may play a major role as a genetic safe guard against progression in patients with PCa.

## Background

Prostate cancer (PCa) has a very high proclivity for metastasizing to bone, with approximately 90% of men with advanced disease having skeletal lesions [1]. Bone is an abundant repository for immobilized growth factors. When osteoclasts absorb bone by secreting protons and proteases, these growth factors are released and provide fertile ground in which tumor cells can grow. Bone-derived growth factors and adhesive molecules secreted by tumor cells can cause modifications in the development and progression of metastatic cancer growth. Indeed, our recent investigation disclosed that a genetic polymorphism in the insulin-like growth factor I gene (*IGF1*) is a strong modifier of PCa progression [2]. Thus, genetic factors involved in the interaction between prostate cancer cells and normal host cells in the bone environment such as osteoclasts and osteoblasts may be a significant determinant in the progression, and bone metastasis, of prostate cancer.

The receptor activator of nuclear factor-kappa B (RANK, also called tumor necrosis factor receptor super family member 11A; TNFRSF11A), its ligand, RANKL (also called TNF ligand super family member 11; TNFSF11), and osteoprotegerin (OPG, also called as TNFRSF11B) are the major regulators of bone metabolism [3,4]. RANKL is a cytokine produced by osteoblasts that stimulates osteoclast activity and inhibits osteoclast apoptosis through binding to its receptor, RANK, which is expressed on osteoclasts and their precursors [3]. OPG, which acts as an inhibitor of osteoclastogenesis by serving as a decoy receptor for RANKL, is a potent inhibitor of osteoclastic bone resorption and has been investigated as a potential therapeutic modality for the treatment of both osteoporosis and tumour-induced bone disease [3-6].

The promoter region of human *OPG *contains various putative transcription factor binding sites that may mediate the stimulation of *OPG *expression by different calciotropic factors [7]. Polymorphisms in this region of *OPG *may contribute to the genetic regulation of bone mass, as suggested by several recent publications [8-13]. Through the inhibition of osteoclast-genesis, OPG has been suggested to be an important modifier of bone metabolism and bone metastasis [3-6]. Consequently, in this study we used a cohort of 361 PCa patients and 195 male controls to investigate the hypothesis that polymorphisms in the promoter region of *OPG *may be genetic modifiers of the development and progression of PCa.

## Methods

### Subjects

A total of 556 native Japanese subjects consisting of 361 PCa patients and 195 male controls were enrolled in our study. Control subjects were selected randomly from a native Japanese population attending a medical check-up at our community hospitals in Akita prefecture. All of the patients with PCa were treated at the Akita University Medical Center and the Kyoto University Hospital from January 1991 to January 2006. All of the PCa patients were diagnosed histologically with specimens obtained from transrectal needle biopsy or transurethral resection of the prostate for voiding symptoms. The prostate specific antigen (PSA) levels of all the controls were measured and men with a PSA level of 4.0 ng/ml or more were omitted from the control group. The pathological grading and clinical stage of PCa were determined according to the Tumor-Node-Metastatic system, the Gleason's histological grading, and the modified Whitmore-Jewett system [14-16]. All pathological grading was based on needle biopsy specimens in stage B-D patients and surgical specimens in stage A patients. We categorized the tumor grading into 3 groups: a high grade group consisting of Gleason 8–10 or poorly differentiated adenocarcinoma, an intermediate grade group consisting of Gleason 5–7 or moderately differentiated adenocarcinoma and a low grade group consisting of Gleason 2–4 or well-differentiated adenocarcinoma [2]. The study was carried out in compliance with the Helsinki declaration and was approved by the institutional review board at Akita University School of Medicine. Written informed consent was obtained from all of the subjects in this study.

### *OPG *genotyping analysis

*OPG *genotyping was analyzed by a polymerase chain reaction restriction fragment length polymorphism (PCR-RFLP) method. Two DNA fragments spanning 178 bp containing the *149 T/C *single nucleotide polymorphism (SNP, rs3134071, 1024 bp upstream of the translation initiation site) and 147 bp containing the *950 T/C *SNP (rs2073617, 223 bp upstream of the translation initiation site) in the *OPG *5' untranslated region were amplified from genomic DNA. The PCR primer sequences were 5'-GTAATCCATGAATGGGACCACACT-3' and 5'-ATCGGAGCTTCCTACGCGCTGA-3', and 5'-GGGGGATCCTTTCCGCCCCA-3' and 5'-GTATCGCCTGCCTTTGATCAGT-3' for the fragment containing the *149 T/C *and *950 T/C *SNPs, respectively. Each PCR reaction was performed as described previously [2]. Digestion of the fragment containing *149 T/C *with *XbaI *resulted in two fragments of 129 and 49 bp containing the *C *allele, and a 178 bp fragment containing the *T *allele. On the other hand, digestion of the PCR products containing *950 T/C *by *HincII *resulted in 2 fragments of 110 and 37 bp with the *C *allele, and a 147 bp fragment with the *T *allele. These genotypes were confirmed by an automated sequencer (ABI PRISM 310 Genetic Analyzer; GMI Inc, Ramsey, MN) with GENESCAN software (Applied Biosystems, Foster City, CA).

### Determination of the serum level of OPG

The serum levels of OPG in 192 male controls and 66 PCa patients were determined using enzyme-linked immunosorbent assays (ELISA, R&D Systems, Minneapolis, MN) according to the manufacturer's protocol. This control group consisted of 192 healthy volunteers, who were recruited in Akita prefecture and whose serum PSA levels were less than 4.0 ng/dl.

### Statistical Analysis

Hardy-Weinberg equilibrium analyses were performed to compare the observed genotype frequencies of each category with the expected ones using a chi-square test (degrees of freedom = 1). The frequency of each allele and the relationship between each genotype were analyzed by chi-square analysis. Possible significant differences in age and serum variables in each genotype-stratified group were assessed by two-way analysis of variance (ANOVA). The age-adjusted odds ratio (aOR) and 95% confidence interval (CI) for the relative risk of PCa and the relationship between the genotype distribution and the tumor grade or stage were determined by multivariate logistic regression analysis with the inclusion of the factor of age. In addition, the gene dosage effect of the *T *allele was assessed by modeling a linear effect on the log odds scale for each *T *allele in a multivariate logistic regression, for example the genotypes *TT*, *TC*, and *CC *were assigned the values "2," "1," and "0," respectively. Survival time was calculated from the date of prostate cancer diagnosis to the day of cancer-specific death, with deaths from other causes excluded. Cancer-specific survival was estimated using the Kaplan-Meier method and the differences in survival were tested using the log rank test. Hazard ratios (HRs) and 95% CIs for cancer death were assessed by the Cox proportional hazard regression model. All of the data were entered into an access database and analyzed using the Excel 2000 and SPSS (version 10.0J; SPSS, Inc.) software. A probability (*P*) of < 0.05 was considered to be statistically significant. The linkage disequilibrium between the two loci was analyzed with LDA software (Linkage Disequilibrium Analyzer, version 1.0, Chinese National Human Genome Center, Beijing, China).

## Results

### Subject Characteristics

The mean age ± SD was 64.6 ± 9.2 years for the controls and 66.8 ± 10.0 for the PCa patients. The controls were significantly younger than the PCa patients (*P *= 0.042). There were 24 stage A patients whose PCa was diagnosed incidentally by specimens removed for Benign prostatic hyperplasia (BPH) treatment. By clinical findings, 184 and 56 patients with PCa were classified into stage B and C disease, respectively. In total, 97 patients were classified as having metastatic PCa; eleven PCa patients had clinical stage D1 disease, while 86 had clinical D2 disease judged by radiological studies. (Table 1).

**Table 1:** *Osteoprotegerin *genotype frequencies in prostate cancer patients (PCa) and control males

**149 *T/C***					
		149 *T/C *Genotype			*C *allele frequency (%)
Study group	Total no.	*CC*	*TC*	*TT*	
PCa^a^	361	4 (1.1)	84 (23.3)	273 (75.6)	12.742
Stage^b^					
A	24	1 (4.2)	5 (20.8)	18 (75.0)	14.583
B	184	1 (0.5)	40 (21.7)	143 (77.8)	11.413
C	56	1 (1.8)	16 (28.6)	39 (69.6)	16.071
D	97	1 (1.0)	23 (23.7)	73 (75.3)	12.886
Organ confined PCa	208	2 (1.0)	45 (21.6)	161 (77.4)	11.778
Extraprostatic PCa	153	2 (1.3)	39 (25.5)	112 (73.2)	14.052
Localized Pca	264	3 (1.1)	61 (23.1)	200 (75.8)	12.689
Metastatic Pca	97	1 (1.0)	23 (23.7)	73 (75.3)	12.887
Grade^c^					
Low/Intermediate	257	1 (0.4)	55 (21.4)	201 (78.2)	11.089
High	104	3 (2.9)	29 (27.9)	72 (69.2)	16.826
					
Control	195	3 (1.5)	37 (19.0)	155 (79.5)	11.026

***950 T/C***					
		*950 T/C *Genotype (%)			*C *allele frequency (%)
Study group	Total no.	*CC*	*TC*	*TT*	

PCa^a^	361	63 (17.4)	162 (44.9)	136 (37.7)	39.889
Stage^b^					
A	24	8 (33.3)	8 (33.3)	8 (33.3)	50
B	184	36 (19.6)	84 (45.6)	64 (34.8)	42.391
C	56	9 (16.0)	30 (53.6)	17 (30.4)	42.857
D	97	11 (10.3)	39 (41.2)	47 (48.5)	30.927
Organ confined PCa	208	44 (21.2)	92 (44.2)	72 (34.6)	43.269
Extraprostatic PCa	153	19 (12.4)	70 (45.8)	64 (41.8)	35.294
Localized Pca	264	53 (20.0)	122 (46.3)	89 (33.7)	43.181
Metastatic Pca	97	11 (10.4)	39 (41.2)	47 (48.4)	30.927
Grade^c^					
Low/Intermediate	257	48 (18.7)	116 (45.1)	93 (36.2)	41.245
High	104	15 (14.4)	46 (44.3)	43 (41.3)	36.538
					
Control	195	29 (14.9)	98 (50.2)	68 (34.9)	40

^a ^Prostate cancer *versus *control, *P *= 0.939 and 0.294 by the chi-square test for *149 T/C *and *950 T/C*, respectively.

^b ^According to the Whitmore-Jewett system. Stage A = T_1a-b_N_0_M_0_, Stage B = T_1c-2_N_0_M_0_, Stage C = T_3–4_N_0_M_0 _and Stage D = T_1–4_N_1_M_0–1 _or T_1–4_N_0–1_M_1_. Localized PCa (Stage A + B + C) *versus *Metastatic PCa (Stage D) *P *= 0.931 and 0.005 by the chi-square test for *149 T/C *and *950T/C*, respectively. Organ confined (Stage A + B) *versus *Extraprostatic PCa (Stage C + D), *P *= 0.346 and 0.021 by the chi-square test for *149 T/C *and *950T/C*, respectively.

^c ^Low grade = well-differentiated carcinoma (WHO) or Gleason score 2–4 carcinoma, Intermediate grade = moderately differentiated carcinoma (WHO) or Gleason score 5–7 carcinoma, High grade = poorly differentiated carcinoma (WHO) or Gleason score 8–10 carcinoma. *C *allele frequency, *P *= 0.884 and 0.520 by the chi-square test for *149 T/C *and *950 T/C*, respectively.

### *OPG *genotypes and risk of PCa

A recent report revealed that the OPG polymorphisms in the promoter region, *149 T/C*, *209 G/A*, and *245 T/G *are in strong linkage disequilibrium [9]. These three polymorphisms have been shown to provide TGT or CAG haplotypes [9]; therefore we investigated the genotype of *149 T/C *among these polymorphisms. In addition, we analyzed another polymorphism, *950 T/C*, which is located 801 bp downstream of the *149 T/C *polymorphism. There is no report showing any linkage disequilibrium between the polymorphism at *950 T/C *and any other surrounding SNPs. We examined the linkage disequilibrium between these two loci. The r^2 ^and D' value of the patients with PCa were 0.1163 and 0.6875, respectively. These results suggested that no tight linkage disequilibrium between the two loci was shown. The frequencies of the *149 T/C *and *950 T/C *genotypes in the PCa and control groups are shown in Table 1. The *OPG *genotype frequency in each group (PCa and control) was in Hardy-Weinberg equilibrium (*P *> 0.05, data not shown). No significant differences in the allele frequencies were found when the PCa patients (*149 T/C*; T:C = 0.87:0.13, *950 T/C*; T:C = 0.60:0.40) were compared with the controls (*149 T/C*; T:C = 0.89:0.11, *950 T/C*; T:C = 0.60:0.40). To evaluate the risk of PCa according to the *OPG *genotype, logistic regression analysis was conducted with an adjustment for age at the time of diagnosis (Table 1). No significant increased risk was observed among the different genotypes of patients with PCa and the controls (*P *= 0.939 and 0.294 for 149 *T/C *and *950 T/C *polymorphisms, respectively).

### *OPG *Genotypes and PCa disease status

Next, we examined the association between the genotypes of *OPG 149 T/C *and *950 T/C *and clinicopathological variables at the time of diagnosis. Regarding the *149 T/C *polymorphism, no significant differences in the allele frequencies were found between low/intermediate and high grades or between low and high stages (data not shown).

Regarding the *950 T/C *polymorphism, no significant differences in the allele frequencies were found between the low/intermediate and high grades (Table 1). However, the frequency of the *CC *genotype in the *950T/C *polymorphism decreased as the tumor stage increased (Table 1). The *TT *and *TC *genotypes were more frequently observed in patients with tumors of a higher stage than those of a lower stage (Table 1). A significant difference in the frequency of the *C *allele was found between the organ-confined PCa patients (stage A and B; T:C = 0.57:0.43) and the extraprostatic PCa patients (stage C and D; T:C = 0.65:0.35, *P *= 0.021), and between the localized PCa patients (stage A, B, and C; T:C = 0.57:0.43) and metastatic PCa patients (stage D; T:C = 0.69:0.31, *P *= 0.005) (Table 1). PCa patients with the *TT *genotype had a 2.76-fold and 2.03-fold increased risk of metastatic and extraprostatic diseases, respectively, compared to patients with the *CC *genotype (Table 2). When the *TT*, *TC*, and *CC *genotypes were assigned the values "2," "1," and "0", respectively, the presence of the *T *allele significantly increased the risk of metastatic disease (aOR = 1.65, 95%CI = 1.17–2.33, *P *= 0.005) and extra prostatic disease (aOR = 1.37, 95%CI = 1.01–1.84, *P *= 0.04) with a gene-dosage effect. However, no significant difference in the genotype frequency was found among the three subgroups of tumor grade (low, intermediate, and high grade; *P *= 0.253).

**Table 2:** Age-adjusted odds ratios according to the *Osteoprotegerin950 T/C *genotype

	Age-adjusted odds ratio (95% CI, *P*) according to the *Osteoprotegerin 950 T/C *genotype^a^		
Study group	*CC*	*TC*	*TT*

Prostate cancer against control	1.00	0.60 (0.345–1.032,0.065)	0.73 (0.418–1.302,0.294)
Tumor stage^b^			
Stage D against Stage A+B+C	1.00	1.72 (0.801–3.698, 0.164)	2.76 (1.289–5.938, 0.009)
Stage C+D against Stage A+B	1.00	1.74 (0.937–3.253, 0.079)	2.03 (1.078–3.843, 0.028)
Tumor grade^b^			
High against Low +Intermediate	1.00	1.27 (0.649–2.498, 0.482)	1.48 (0.750–2.947, 0.256)

a These data were adjusted for age.

b The tumor stage and grade systems are the same as those in Table 1.

### Patient prognosis with metastatic PCa (Stage D) and *OPG *genotypes

**Figure 1:**
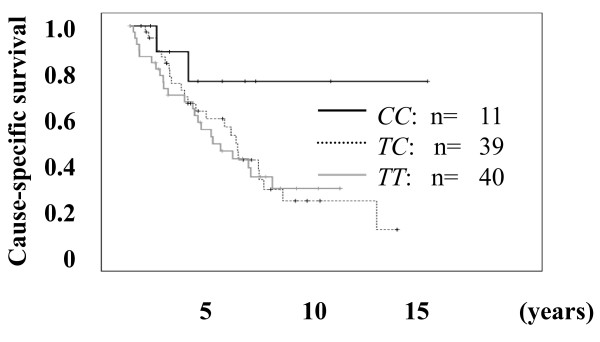
**Comparison of the outcome of patients with PCa and the *950 T/C OPG *genotype**. The differences in cause-specific survival of the patients with the *CC *genotype versus the *TC *and *TT *genotypes were an OR of 2.938 and 3.018 (*P *= 0.087 and 0.082, respectively).

Next, we investigated the association between the clinical variables and *OPG 149 T/C *and the *950 T/C *genotype in patients with metastatic PCa (Stage D) after the initial diagnosis. Among the patients with metastatic PCa, the outcome and the serum variables of 90 patients were available for this study. Regarding the *149 T/C *polymorphism, no significant differences in the allele frequencies were found between the clinical variables of these patients and the genotype (data not shown). Regarding the *950 T/C *polymorphism, age, initial PSA, hemoglobin, alkaline phosphatase (ALP), and the biopsy histological grade were not significantly different among the *CC*, *TC*, and *TT *genotypes (Table 3). However, although the difference was not significant, the cause-specific survival of patients with the *CC *genotype was better than that of patients with the *TC *or *TT *genotype (OR = 2.938 and 3.018 for *TC *and *TT *genotypes compared with the *CC *genotype, *P *= 0.087 and 0.082, respectively. Figure 1). In addition, according to the multivariate Cox proportional hazard regression analyses, the *T *allele of the *OPG 950 *T/C polymorphism was an independent risk factor predicting survival with HRs of 2.157 (95% CI = 1.072 – 4.341, *P *= 0.031, Table 4) compared to the *C *allele. These results also suggested that the variant *C allele *of *950 T/C *in the *OPG *promoter may have some protective effect against the progression of PCa.

**Table 3:** Association between the clinical variables of patients with metastatic prostate cancer (Stage D) and the *OPG 950T/C *genotype

	*Osteoprotegerin 950T/C *genotype			
	*CC*	*TC*	*TT*	*P*^3^
	n = 11	n = 39	n = 40	
Age^1^	74 (69–79)	75 (68–81)	72 (65–76)	0.375
Histological grade^2^				
Low/Intermediate	4 (9%)	18 (39%)	24 (52%)	0.641
High	7 (16%)	21 (48%)	16 (36%)	
Clinical variables at diagnosis^1^				
PSA (ng/ml)	199 (66.4–296)	257 (37.3–860)	116 (30.0–267)	0.270
Hemoglobin (g/dl)	12.8 (12.2–13.1)	12.7 (11.1–13.7)	13.2 (12.0–14.6)	0.194
ALP (IU/ml)	189 (175–468)	232 (177–360)	201 (138–343)	0.659

^1^Values represent the median (inter-quartile range).

^2^Values represent the number (%).

^3^Analysis of variance or chi-square test.

Abbreviations: PSA; prostate specific antigen, ALP, alkaline phosphatase.

**Table 4:** Multivariate Cox proportional hazard regression analysis of predicting factors for cancer-specific survival of metastatic prostate cancer patients (Stage D)

	HR	Cancer Specific Survival 95% CI	*P*
PSA (ng/ml)			
≥260	1.984	0.756–5.210	0.164
< 260			
hemoglobin (g/dl)			
Abnormal (< 11.7)	2.632	1.140–6.077	0.023
Normal (≥11.7)			
ALP (IU)			
Abnormal (≥ 282)	2.557	0.994–6.574	0.051
Normal (< 282)			
Grade			
High	3.251	1.473–7.175	0.004
Low/Intermediate			
*OPG*			
*T allele *dosage	2.157	1.072–4.341	0.031

Abbreviations: HR; hazard ratio, 95% CI; 95% confidence interval, PSA; prostate specific antigen, ALP, alkaline phosphatase, *OPG*; *osteoprotegerin 950 T/C genotype*

### Serum level and genotypes of OPG in healthy controls and patients with PCa

**Figure 2:**
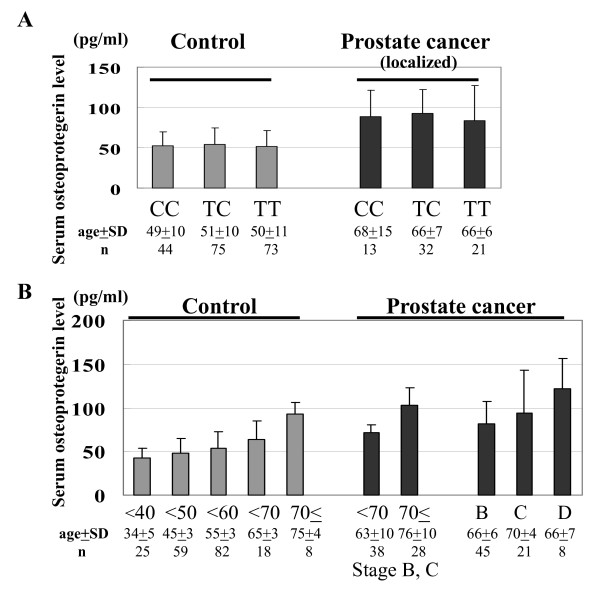
**The serum OPG levels in healthy controls and patients with PCa**. (A) Comparison of the serum OPG levels and the *950 T/C *genotype in 192 healthy controls and 66 localized PCa patients. (B) Comparison of the serum OPG level by age and disease status (stage B, C, and D) in healthy controls and patients with PCa. *Age represents mean ± standard deviation.

We hypothesized that the promoter activity of *OPG *would reflect the OPG serum level. Consequently, we investigated the association between the serum level of OPG and *149 T/C *and *950 T/C *genotypes in 192 normal controls and 74 PCa patients whose serum was available for this study. The mean serum levels of OPG for all of the normal controls, the patients with localized PCa and those with metastatic PCa were 52.6 ± 19.7 pg/ml (*n *= 192; median 48.7, quartile 39.3–60.4), 84.2 ± 32.7 pg/ml (*n *= 66; median 82.1, quartile 64.4–101), and 122.2 ± 34.4 pg/ml (*n *= 8; median 132, quartile 105–141.7), respectively. The serum level of OPG in patients with PCa was significantly higher than that of healthy controls (*P *= 3.50 × 10^-22^); however, the age of the patients with PCa was also significantly higher than that of the healthy controls (*P *= 1.47 × 10^-13^). Comparison of the OPG serum level in healthy controls and patients with PCa was therefore not conclusive. We also investigated the relationship between the OPG serum level and *the 149 T/C *and the *950 T/C *genotypes in healthy controls and patients with PCa; no significant association was found (Figure 2A).

### OPG serum level by age and disease status in healthy controls and patients with PCa

We investigated the association between the OPG serum level and the age and disease status of the healthy controls and patients with PCa. We detected a significant increase in the serum levels of OPG with age in the healthy controls, as well as in patients with non-metastatic PCa (stage B and C, Figure 2B). In addition, there was no significant difference between the age-matched serum OPG levels of the healthy controls and patients with non-metastatic PCa. The serum level of OPG in age-matched patients with metastatic PCa (stage D2) was significantly higher than that of patients without metastatic disease and healthy controls (*P *= 0.012 and 0.005, respectively. Figure 2). These results suggest that aging and bone metastasis have a major effect on the serum level of OPG rather than PCa.

## Discussion and conclusion

Over a hundred years ago, Stephen Paget enunciated the "seed and soil" hypothesis in which seeds of metastatic cancer cells preferentially settle in the soil of the bone matrix, where there is an abundant repository for immobilized growth factor [17]. In this study, we hypothesized that the host's bone metabolism may modify disease development and progression. Among many factors that play important roles in bone metabolism, OPG is a major factor in osteoclastgenesis and is known as osteoclast inhibitory factor [3,4]. In this study, no significant differences in the allele frequencies of *149 T/C *polymorphism were found in the clinical and pathological variables in the patients with PCa. However, this study demonstrated that the *C *allele of the *950 T/C *polymorphism in the *OPG *promoter region decreased the risk of advanced PCa with a gene-dosage effect. The *C *allele was found at a significantly lower frequency in patients with metastatic (stage D) and extraprostatic PCa (stage C+D) than in patients with localized (stage A+B+C) and organ confined PCa (stage A+B) (Table 2). In addition, the analysis of metastatic PCa patients (Stage D) revealed that the *T *allele of the OPG *950 T/C *polymorphism was an independent risk factor predicting survival compared to the *C *allele by Cox proportional hazard regression analyses (*P *= 0.031). Although the *950 T/C *is not a putative transcription factor binding site, an altered secondary structure may cause differences in *OPG *mRNA expression. Therefore, these results suggest that the *T/C *allele of the *950 T/C *polymorphism functions as a modifier of OPG expression and has some effect on metastasis and the spread of PCa cells. Alternatively, it is possible that this polymorphism is in linkage disequilibrium with other SNP(s) that alters OPG function.

Although conflicting results have been documented [8-10], the genotype of the *950 T/C *polymorphism has been reported to be independently associated with a subset of osteoporosis or bone marrow density (BMD). Vidal et al. reported that the *T *allele of the *950 T/C *polymorphism was associated with low BMD, although statistical significance was not reached [11]. Arko et al. also revealed that the homozygote *CC *genotype of the *950 T/C *polymorphism had the highest BMD of all of the anatomical sites [12]. In addition, Ohmori et al. demonstrated that the *TT *genotype had a significantly lower BMD than the *TC *and *CC *genotypes by parametric and non parametric analysis [13]. Interestingly, they used healthy DNA samples from the same prefecture, Akita in Japan. In addition, recent studies reported that the *C *allele *950 T/C *polymorphism contributed to calcification of the vascular system and the osseous tissue [18-20].

Instead of measuring the expression level of OPG in the bone tissues, we compared the serum level of OPG for each of the genotypes; however, there was no difference among these genotypes either in the healthy controls or the patients with PCa. The serum level of OPG was significantly higher in patients with bone metastasis than those without bone metastasis; this is in agreement with previous reports [21-23]. In addition, we found that the serum level of OPG in the older individuals was significantly higher than that of the younger ones. Our results support previous findings that the serum levels of OPG increase with age in relatively large healthy adult Caucasian populations [24,25]. In contrast, Lipton et al. reported that serum OPG values did not differ significantly by age, although they analyzed male and female healthy control samples together [26]. The relationship between age and serum OPG warrants further investigation because healthy controls have a tendency to be younger than patients with cancer, and patients with advanced stage disease are usually older than those with early stage cancer. Cao et al. demonstrated that *OPG *mRNA levels in bones of the adult male mouse were lower than those in young mice [27]. These results indicate that the serum levels of OPG do not precisely reflect the expression profile or OPG level in the local bone environment. Therefore, we could not confirm whether each *950 T/C *polymorphism genotype had a considerable effect on *OPG *expression.

In conclusion, the *OPG 950 T/C *polymorphism was not associated with susceptibility to PCa in Japanese men. However, the presence of the variant *C *allele may have a considerable protective effect against metastasis or advanced disease status in patients with PCa. This effect is not associated with a biologically less aggressive (low grade) tumor, but may be due to the modification of cancer progression and metastasis through the host's bone metabolism. In order to clarify the role of OPG in the progression of PCa, further molecular studies are warranted.

## Competing interests

The authors declare that they have no competing interests.

## Authors' contributions

FN, TY, NT, TK, and SI carried out the molecular genetics studies, participated in the sequence alignment, drafted the manuscript. TI and MS performed the ELISA. ZM, YH, and SS performed the statistical analysis. TY, OO, and TH conceived of the study, and participated in its design and coordination. All authors read and approved the final manuscript.

## Pre-publication history

The pre-publication history for this paper can be accessed here:


